# GLAGC: Adaptive Dual-Gamma Function for Image Illumination Perception and Correction in the Wavelet Domain

**DOI:** 10.3390/s21030845

**Published:** 2021-01-27

**Authors:** Wenyong Yu, Haiming Yao, Dan Li, Gangyan Li, Hui Shi

**Affiliations:** 1 School of Mechanical Science and Engineering, Huazhong University of Science and Technology, Wuhan 430074, China; ywy@mail.hust.edu.cn (W.Y.); u201812016@hust.edu.cn (H.Y.); u201810878@hust.edu.cn (D.L.); 2 School of Mechanical and Electronic Engineering, Wuhan University of Technology, Wuhan 430074, China; ligy@hust.edu.cn

**Keywords:** adaptive gamma correction, image illumination correction, wavelet transforms, image enhancement, illumination perception

## Abstract

Low-contrast or uneven illumination in real-world images will cause a loss of details and increase the difficulty of pattern recognition. An automatic image illumination perception and adaptive correction algorithm, termed as GLAGC, is proposed in this paper. Based on Retinex theory, the illumination of an image is extracted through the discrete wavelet transform. Two features that characterize the image illuminance are creatively designed. The first feature is the spatial luminance distribution feature, which is applied to the adaptive gamma correction of local uneven lighting. The other feature is the global statistical luminance feature. Through a training set containing images with various illuminance conditions, the relationship between the image exposure level and the feature is estimated under the maximum entropy criterion. It is used to perform adaptive gamma correction on global low illumination. Moreover, smoothness preservation is performed in the high-frequency subband to preserve edge smoothness. To eliminate low-illumination noise after wavelet reconstruction, the adaptive stabilization factor is derived. Experimental results demonstrate the effectiveness of the proposed algorithm. By comparison, the proposed method yields comparable or better results than the state-of-art methods in terms of efficiency and quality.

## 1. Introduction

Uneven or insufficient illumination will cause the contrast of an image to be too low, making it difficult to observe the details of the image. We usually pursue for the enhancement results that local variation is obvious while the global variation is in accordance with the original intensity, which is denoted as naturalness preservation. Researchers have proposed many enhancement methods to make these images have a more pleasing visual effect or to obtain high-visibility effects.

Pixel modulation schemes, such as statistics-based method histogram equalization (HE), directly adjust the pixel intensity of the image to achieve enhancement. This kind of method may cause artifacts and the loss of naturalness. The nonlinear gamma correction approach uses different mapping curves to achieve excellent performance in complex lighting conditions [1], but the parameters need manual design with prior knowledge, and the spatial information is not considered [2] when operating on each pixel.

Converting pixel information to other domains can yield more internal information of the image, such as discrete Fourier transform, discrete cosine transform (DCT), and discrete wavelet transform (DWT). These solutions achieve effects through filters in the frequency domain and reconstruction in the spatial domain, such as homomorphic filtering, which may result in the loss of potentially useful visual cues [3].

To conduct an analysis from the perspective of the image physical process, Retinex theory is proposed to simulate the relationship between the illumination component and the reflection component of an image [4,5]. A series of methods were derived, such as the single-scale Retinex (SSR) algorithm [6] and multiscale Retinex (MSR) algorithm [7], to enhance the image details. However, the naturalness of the images may be destroyed, and it is unreasonable to treat only the reflectance layer as the enhanced image [8].

As a spatial-frequency analysis tool, the DWT is applied to decompose and enhance image features at different resolutions. It has been utilized by researchers in the fields such as image resolution enhancement [9] and image denoising [10].

Existing methods have difficulty balancing brightness correction, naturalness preservation, color restoration, and algorithmic efficiency. A simple but efficient algorithm for image illuminance perception and correction in the wavelet domain is proposed in this paper. The DWT is used to separate the illuminances in the low-frequency subband, which will be enhanced by adaptive gamma correction considering both the spatial and statistical characteristics of the image. For naturalness preservation, adaptive punishment adjustment is applied for the high-frequency subband. Finally, a stabilization factor is designed for color restoration so that the extralow illumination can be corrected with noise suppression. To the best of our knowledge, no work has proposed an adaptive dual-gamma correction method in the wavelet domain.

The rest of the paper is organized as follows: Section 2 provides a brief discussion of related works. Section 3 presents the detailed process of the proposed method. In Section 4, the superiority of the proposed method is supported by experimental results and relevant evaluation with state-of-the-art models. Finally, the conclusions are presented in Section 5.

## 2. Related Works

To solve the problems mentioned above, improvements have been proposed in earlier works. There are several variations of the HE method, such as contrast-limited adaptive histogram equalization (CLAHE) [11] and brightness-preserving bi-histogram equalization (BBHE) [12]. In the frequency domain, improved methods such as illuminance normalization based on homomorphic filtering [3], color image enhancement by compressed DCT [13], and the alpha-root method based on the quaternion Fourier transform [14] are proposed. The following methods are comparable to our work:

- Improved gamma correction: For parameter adjustment, some adaptive methods are derived, such as adaptive gamma correction based on cumulative histogram (AGCCH) [15], adaptive gamma correction to enhance the contrast of brightness-distorted images [16], adaptive correction with weight distribution (AGCWD) method [17], and a 2-D adaptive gamma correction method [18], which takes into account the variable brightness map of image spatial information while excessive contrast enhancement may occur. In addition, few methods consider both local and global enhancement, and overenhancement sometimes appears in some portions of the image.
- Retinex-based model: Fu et al. [19] proposed a simultaneous illumination and reflectance estimation (SIRE) method to preserve more image details when estimating the reflection intensity. Wang [20] used Retinex theory to construct an image prior model and used a hierarchical Bayesian model to estimate the model parameters and achieved good results. Cheng [21] proposed a nonconvex variational Retinex model to improve the brightness while maintaining the texture and naturalness of an image. These models based on Retinex theory can achieve pleasing reflection separation through iterations. However, the algorithms are time-consuming and may limit their practical applications. Low-light image enhancement via well-constructed illumination map estimation (LIME) was proposed by Guo [2]. Oversaturation in some portion of an image usually occurs.
- Combining the wavelet transform approach: By introducing the wavelet transform, a nonlinear enhancement function was designed based on the local dispersion of the wavelet coefficients [21]. Zotin [22] proposed an algorithm combining the MSR algorithm with the wavelet transform algorithm and achieved a better correction effect in terms of efficiency. A dual-tree complex wavelet transform for low-light image enhancement was proposed in [23]. However, it is unreasonable to utilize only the low-frequency subband for illumination enhancement. The image edges will appear jagged after transformation according to our experiments.

## 3. Proposed Method: GLAGC

### 3.1. Algorithm Scheme

**Figure 1:**
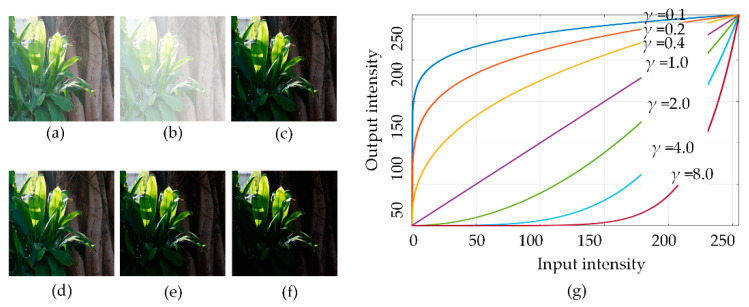
An example of gamma correction, the enhanced images with different parameters *γ*. (**a**) Original image. (**b**) *γ* = 0.1. (**c**) *γ* = 0.3. (**d**) *γ* =0.8. (**e**) *γ* = 1.2. (**f**) *γ* = 1.5. (**g**) The curve along with different parameters *γ*.

Gamma correction [17] is a common method for illumination enhancement and is defined as:

(1) $$I^{\prime }=I_{\max}\times (\frac{I}{I_{\max}}){}^{\gamma }$$

where *I*′ is the corrected image, *I*_max_ is the maximum intensity value of the original image, *I* is the original image, and *γ* is the parameter. For different values of *γ*, the resulting image has different enhancement results, as shown in Figure 1. When *γ* < 1, low-intensity pixels will be increased more than high-intensity pixels. When *γ* > 1, the opposite effect is generated. When *γ* = 1, the input and output intensities are equal.

The limitations of the conventional gamma correction method are obvious: (1) The selection of the parameters requires experience. (2) Spatial information such as uneven lighting of the image is not considered. (3) The overall illumination cannot be perceived, and overexposure sometimes occurs.

For this reason, a novel adaptive gamma correction method, called global statistics and local spatial adaptive dual-gamma correction (GLAGC), is proposed in this section. First, the V component of from HSV model of the input image is converted to the logarithmic domain. Through the DWT, the illumination information of the image is obtained from the low-frequency subband *LL*. The dual-gamma correction *γ*(*θ*_[*χ*,*σ*]_) based on spatial and statistical information is applied to subband *LL*:

(2) $${LL}^{\prime }=I_{MAX}\times {\left(\frac{LL}{I_{MAX}}\right)}^{\gamma (\theta_{\left[\chi ,\sigma \right]})}$$

where *I_MAX_* is the maximum pixel value of the *LL* subband and *LL*′ is the corrected low-frequency subband.

**Figure 2:**
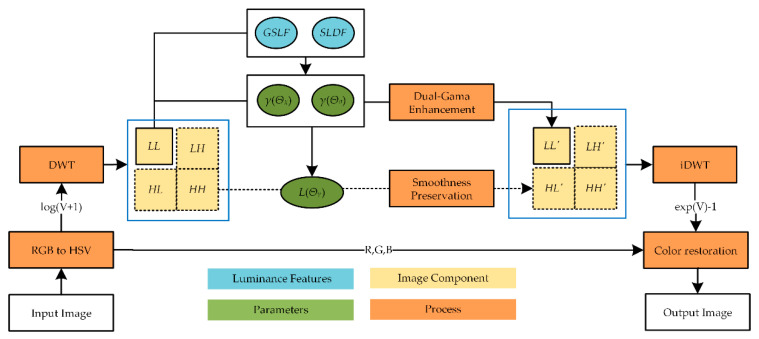
Flowchart of GLAGC method.

For naturalness preservation, adaptive punishment adjustment is applied in the *LH, HL*, and *HH* subbands. Then, the corrected V component is obtained through the inverse wavelet transform. Finally, the enhanced image is reconstructed by converting it to the RGB color space through color restoration. The process flow of the proposed image enhancement method is shown in Figure 2.

### 3.2. Luminance Extraction in the Wavelet Domain

According to Retinex theory, an image can be expressed as the multiplicative combination of the reflection intensity and the illumination brightness, namely:

(3) S(x,y)=L(x,y)×R(x,y)

where *S*(*x*, *y*) is the pixel information of the image and *R*(*x*, *y*) is the reflection intensity, reflecting the surface properties of the object color, texture, etc. that correspond to the high-frequency information of the image; *L*(*x*, *y*) is the environmental illumination, which depends on the external lighting conditions and corresponds to the low-frequency information of the image. Since the operation in the logarithmic domain is closer to the visual characteristics perceived by the human eye, the image is converted to the logarithmic domain to obtain the additive combination of reflection intensity and illumination brightness:

(4) s(x,y)=l(x,y)+r(x,y)

where *s*(*x*, *y*) = log(*S*(*x*, *y*)), *r*(*x*, *y*) = log(*R*(*x*, *y*)), and *l*(*x*, *y*) = log(*L*(*x*, *y*)). To obtain the illumination component *l*(*x*, *y*), a center/surround Retinex method such as the SSR algorithm uses the convolution of the Gaussian function and the image *s*(*x*, *y*):

(5) l(x,y)=s(x,y)∗G(x,y)

(6) $$G(x,y)=k\times e^{\frac{-(x^{2}+y^{2})}{c^{2}}}$$

where * is a convolution operation, *G*(*x*, *y*) is the Gaussian convolution function, ∬*G*(*x*, *y*) = 1; *c* is the scale factor, and *k* is the normalization constant. The MSR algorithm uses multiscale Gaussian functions:

(7) $$l(x,y)=\sum_{n=1}^{N}\varpi_{n}\times \{s(x,y)*G_{n}(x,y)\}$$

where *G_n_* (*x*, *y*) is the Gaussian function of the *n*-th scale and the weight *ϖ_n_* satisfies $\sum_{n=1}^{N}\varpi_{n}=1$.

State-of-the-arts methods like SSR and MSR obtain illumination feature by using Gaussian convolution within certain perception domain. Gaussian convolution will cause computational complexity. Moreover, the neighboring pixel information also includes the edge of the image, texture and other redundant details that do not contribute to the illuminance features. This paper takes a different approach that illumination extraction is conducted in the low frequency sub-band of the wavelet domain, while the details of image are extracted in high frequency sub-band. The DWT [24] of a digital image *f* (*x*, *y*) can be expressed as:

(8) $$W_{\phi }(j_{0},m,n)=\frac{1}{M\times N}\sum_{x=0}^{M-1}\sum_{y=0}^{N-1}f(x,y)\varphi_{j_{0},m,n}(x,y)$$

(9) $$W_{\psi }^{i}(j_{0},m,n)=\frac{1}{M\times N}\sum_{x=0}^{M-1}\sum_{y=0}^{N-1}f(x,y)\psi_{j_{0},m,n}^{i}(x,y)\;\;\;\;\;\;\;\;\;\;i\in \{H,V,D\}$$

where *φ* is the scale function; *ψ* is the wavelet function; (*M*, *N*) is the size of the image; *j*_0_ is the initial scale; *W_ϕ_* (*j*_0_, *m*, *n*) is the low-frequency wavelet coefficient, which is an approximation of *f* (*x, y*); index *i* identifies the directional wavelets in terms of values of *H*, *V*, and *D*; and *W^i^_ψ_*(*j*, *m*, *n*) is the high-frequency wavelet coefficient. When the scale *j* ≥ *j*_0_, it means the horizontal, vertical, diagonal details in three directions.

The DWT uses low-pass and high-pass filters to decompose the pixel information of the image into 4 subbands, namely, *LL*, *LH*, *HL*, and *HH*. *LL* denotes the low-pass subband, and *LH*, *HL*, and *HH* denote the vertical, horizontal and diagonal subbands, respectively, where:

(10) $$LL=W_{\phi }(j_{0},m,n)$$

(11) $$LH,HL,HH=W_{\psi }^{i}(j,m,n),i\in \{H,V,D\}$$

From the perspective of the frequency domain, the high-frequency subband after applying the wavelet transform contains only detailed information, such as the edge of the image object, which ensures that the illumination component of the image is included in the low-frequency subband *LL*. Therefore, the illumination of the image can be corrected by using only the low-frequency subband. After illuminance correction in the low-frequency subband, we can use the inverse wavelet transform to obtain the reconstructed image:

(12) $$O(x,y)=iDWT\{W_{\phi }^{\prime }(j_{0},m,n),W_{\psi }^{i}{}^{\prime }(j,m,n)\}$$

where *W*′*_ϕ_*(*j*_0_, *m*, *n*), *W_ψ_^i^*′(*j*, *m*, *n*) is the corrected coefficient, *iDWT*{} represents the inverse wavelet transform, and *O*(*x*, *y*) denotes the corrected image. Next, the proposed adaptive dual-gamma correction method for low-frequency subband *LL* based on the extracted illumination features is described.

### 3.3. Local Spatial Adaptive Gamma Correction (LSAGC)

A spatial luminance distribution feature (SLDF) is proposed, which is defined as:

(13) $$SLDF(x,y)=\sum_{n=1}^{N}\varpi_{n}\times \{LL*G_{n}(x,y)\}$$

where *SLDF*(*x, y*) obtains the pixel neighborhood information by applying a convolution operation to estimate the local spatial distribution of the image′s illumination.

**Figure 3:**
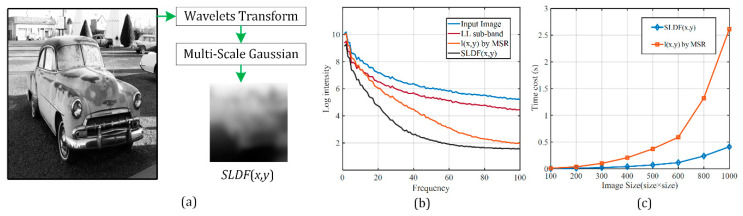
(**a**) Illuminance feature *SLDF*(*x*,*y*) extraction. (**b**) Frequency domain analysis. (**c**) Time-consumption of *SLDF*(*x*,*y*) and MSR.

Figure 3 illustrates the *SLDF*(*x*, *y*) of an image, its frequency domain analysis diagram and time-consumption analysis of our method and MSR. In Figure 3b, the Y-axis denotes the average Fourier log intensity [25] of the image, and the X-axis denotes the frequency. In Figure 3c, the Y-axis denotes the average time consumption of illumination extraction, and the X-axis denotes the image size.

From Figure 3b,c we found:

(1) The frequency components of the illumination extracted by the MSR algorithm are included in the frequency components of the *LL* subband, which means that the illumination of the image can be extracted only in the *LL* subband.
(2) As the frequency increases, the amplitude of *SLDF* (*x*, *y*) attenuates faster. This property is helpful in preserving the image details from the perspective of the local illumination characteristics.
(3) For images with common image sizes, the proposed *SLDF* illumination extraction time is much less than that of the MSR algorithm, and the benefit of the *SLDF* scheme compared with the MSR algorithm increases as the image size increases.

The uneven spatial distribution of the image illuminance appears as overexposure or underexposure in certain areas. The proposed local spatial adaptive gamma correction (LSAGC) method is applied to *LL*, which is defined as:

(14) $$\gamma (\Theta_{\chi })={\left(M_{SLDF}/I_{MAX}\right)}^{\sigma }$$

(15) $$\sigma =2\times [M_{SLDF}-SLDF(x,y)]/I_{MAX}$$

where *M_SLDF_* is the average of *SLDF* (*x*, *y*) and σ is the difference between the brightness of a certain pixel and the average intensity. When the spatial brightness of the image is evenly distributed, (*M_SLDF_*/*I_MAX_*) is close to 1, and the *γ*(*Θ_χ_*) correction ability becomes weak. When *SLDF* (*x*, *y*) is greater than *M_SLDF_*, strong illumination appears, which makes *σ* < 0; thus, the illumination will be reduced by (14). In contrast, the brightness of the dark area will be increased, so uneven lighting is improved through adaptive correction. Applying *γ*(*Θ_χ_*) to *LL*:

(16) $$LL_{LS}=I_{MAX}\times {\left(\frac{LL}{I_{MAX}}\right)}^{\gamma (\Theta_{\chi })}$$

where *LL_LS_* indicates the low-frequency subband corrected by LSAGC.

**Figure 4:**
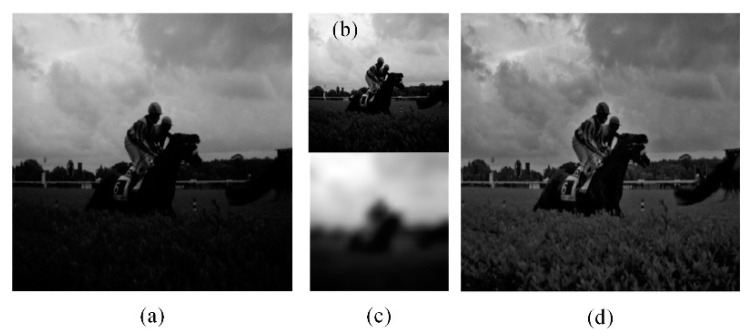
Result of the LSAGC method. (**a**) Original image. (**b**) *LL*. (**c**) *SLDF* (*x*, *y*) of *LL*. (**d**) Reconstructed image after applying LSAGC.

Figure 4 illustrates the *LSAGC* results. An image with uneven illumination is shown in Figure 4a. The *LL* subband obtained by wavelet transform is shown in Figure 4b, and *SLDF* (*x*, *y*) is shown in Figure 4c. The reconstructed image by *iDWT*{*LL_LS_*,*W_ψ_^i^* (*j*, *m*, *n*)} is shown in Figure 4d.

It can be seen from Figure 4d that although the uneven spatial illumination distribution of the image has been corrected, the overall brightness is still low, resulting in unclear details, such as the human face and horse body. Further, more overall luminance correction is required.

### 3.4. Global Statistics Adaptive Gamma Correction (GSAGC)

#### Global Statistical Luminance Feature (GSLF)

**Figure 5:**
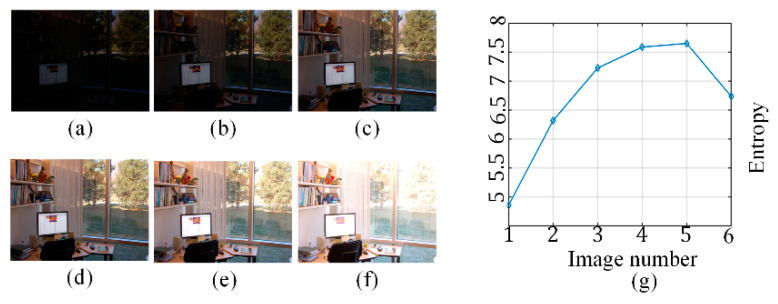
Comparison of the entropy among different images. (**a**–**f**) Images with different luminance values. (**g**) Entropy comparison.

The information entropy of the image represents the aggregation feature of the grayscale value distribution, which is defined as:

(17) $$Entropy=-\sum_{i=0}^{255}p_{i}\times \log2p_{i}$$

where *p_i_* is the probability of a certain grayscale value. Figure 5a–f show images of different luminance conditions with their grayscale distribution histograms. When the image is properly exposed, the grayscale distribution histograms show uniform distributions, and their information entropy is the largest, as shown in Figure 5g.

The probability density function (*pdf*) and cumulative distribution function (*cdf*) of the image are defined as follows:

(18) $$pdf(i)=n_{i}/N$$

(19) $$cdf(i)=\sum_{k=0}^{i}pdf(k)$$

where *i* is the pixel intensity, *n_i_* is the number of pixels with intensity *i*, and *N* is the total number of pixels in the image. According to the maximum discrete entropy theorem, the image with the largest entropy has a uniformly distributed grayscale histogram, and its *cdf*(*i*) has linear characteristics, namely:

(20) ∀i,pdf(i)=c,cdf(i)=c×i

Here, (20) is converted to the logarithmic domain:

(21) cdf(l)=exp(l)

where *l* = *log*(*c* × *i*) and *c* is a constant. In our research, the *cdf*(*l*) of subband *LL* of the image with the largest entropy in the logarithmic domain through wavelet decomposition is constructed as an intensity-guided distribution (*IGD*) function. It plays a guiding role in image illumination correction. The *IGD* function is defined as:

(22) $$IGD(l)=\frac{1}{\exp(I_{max})}\exp(l),l\in (0,I_{max})$$

The *pdf*(*l*) of the subband *LL* is normalized as:

(23) $$pdf_{norm}(l)=\frac{pdf(l)-pdf_{\min}}{pdf_{\max}-pdf_{\min}}$$

where *pdf*_max_ and *pdf*_min_ are the maximum and minimum values of the image *pdf*, respectively. According to the difference between *cdf*(*l*) of the input image and *IGD*(*l*) of the ideal image with the largest entropy, *pdf_GW_*(*l*) and *cdf_GW_*(*l*) are designed as follows:

(24) $$pdf_{GW}(l)=pdf{\left(l\right)}_{norm}^{1-\{1-[cdf(l)-IGD(l)]\}}$$

(25) $$cdf_{GW}(l)=\sum_{k=0}^{l}pdf_{GW}(K)$$

**Figure 6:**
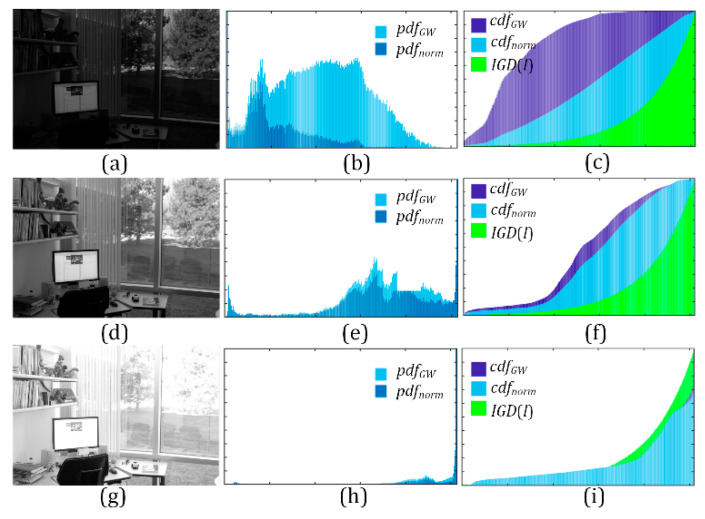
(**a**,**d**,**g**) Original image. (**b**,**e**,**h**) The comparison of *pdf_norm_*(*l*) and *pdf_GW_* (*l*). (**c**,**f**,**i**) The comparison of *cdf*(*l*), *cdf_GW_* (*l*) and IGD(l).

Figure 6 demonstrates three different images of the same scene, which appear underexposed in Figure 6a, properly exposed in Figure 6d and overexposed Figure 6g. A comparison of *pdf_norm_* (*l*) and *pdf_GW_*(*l*) is shown in Figure 6b,e,h, respectively. The relationship among *cdf*(*l*), *cdf_GW_*(*l*) and *IGD*(*l*) is shown in Figure 6c,f,i, respectively. The luminance distribution can be estimated according to the difference between *cdf_GW_*(*l*) and *cdf*(*l*). For an underexposed image, the area enclosed by *cdf*(*l*) and the X-axis is far larger than the area enclosed by *cdf_GW_*(*l*) and the X-axis. For a properly exposed image, the area enclosed by *cdf*(*l*) and the X-axis is close to the area enclosed by *cdf_GW_* (*l*) and the X-axis. For an overexposed image, the area enclosed by *cdf*(*l*), and the X-axis is close to the area enclosed by *cdf_GW_* (*l*) and the X-axis but smaller than that of *IGD*(*l*).

For the correction of the overall illumination brightness of an image, a global statistical luminance feature (*GSLF*) is designed to evaluate the difference between *cdf*(*l*) and *cdf_GW_*(*l*), which is defined as:

(26) $$GSLF=\sum \left|\frac{cdf_{GW}(l)-cdf(l)}{cdf(l)}\right|$$

In our research, a global statistics adaptive gamma correction (GSAGC) method is proposed as *γ*(*Θ_σ_*), which is applied to subband *LL*:

(27) $$LL_{GS}=I_{MAX}\times {\left(\frac{LL}{I_{MAX}}\right)}^{\gamma (\Theta_{\sigma })}$$

where *LL_GS_* indicates the corrected low-frequency subband by GSAGC. Through a training set containing images with various illuminance conditions, the relationship between *γ*(*Θ_σ_*) and the *GSLF* will be estimated.

Training Datasets: This article has established an image dataset collected from related works [2,15,18,20,21,22,26] containing different luminance conditions, including underexposure, proper exposure, and uneven exposure.

**Figure 7:**
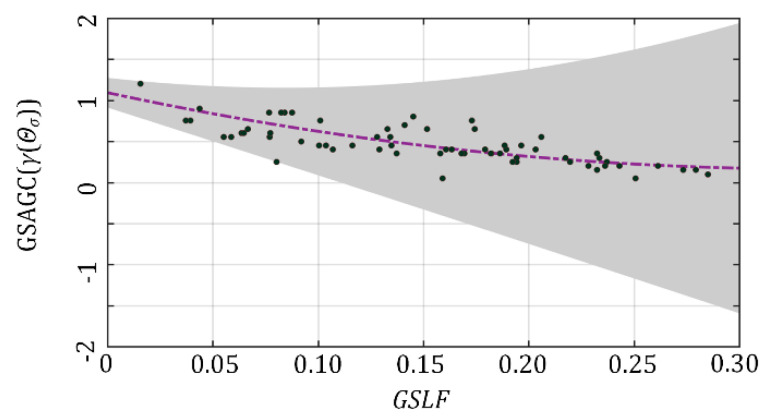
Regression curve of *γ*(*Θ_σ_*) and *GSLF*. Confidence interval is 95%.

Loss Function: To judge whether the overall illumination intensity of an image satisfies the maximum entropy criterion, we introduce the information entropy loss function to obtain the global statistics adaptive gamma *γ*(*Θ_σ_*), namely:

(28) $$Loss_{Entropy}(D)=-\sum_{\alpha =1}^{N}Entropy(D_{\alpha })$$

(29) $$D_{\alpha }=iDWT\{LL_{GS},W_{\psi }^{i}(j,m,n)\}$$

where *D* is the training dataset, *D_α_* is a reconstruction sample, and *N* is the number of samples in the training dataset. When the information entropy loss function of the reconstructed images is minimized, the regression curve indicating the relationship between *γ*(*Θ_σ_*)**^1×1×N^** and *GSLF***^1×1×N^** is obtained in Figure 7:

(30) $$\gamma (\Theta_{\sigma })=8.224\times GSLF^{2}-5.534\times GSLF+1.093$$

According to the above, the proposed adaptive dual-gamma correction function, GLAGC, which takes into account the *γ*(*Θ_χ_*) by LSAGC and the *γ*(*Θ_σ_*) by GSAGC, is given as:

(31) $$\gamma (\Theta_{\left[\chi ,\sigma \right]})=\gamma (\Theta_{\chi })\times \gamma (\Theta_{\sigma })$$

### 3.5. Smoothness Preservation

**Figure 8:**
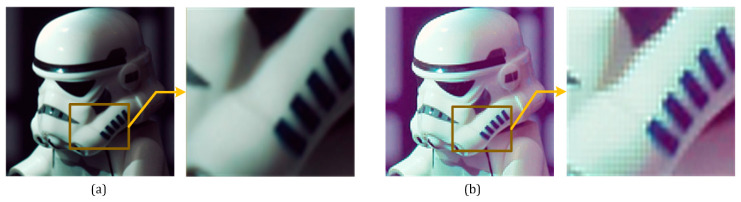
(**a**) Original image. (**b**) Reconstructed image by (29).

Since GLAGC is adopted in the low-frequency subband *LL* in the wavelet domain, the high-frequency subband needs to be adjusted correspondently. Otherwise, jaggedness will appear at the image edges after inverse wavelet transformation, as shown in Figure 8. Thus, we introduce a smoothness adjustment to the wavelet high-frequency subband, denoted by:

(32) $$W_{\psi }^{i}=L(\Theta_{\gamma })\times W_{\psi }^{i}$$

where *W_ψ_^i^* is the high-frequency wavelet coefficient and *L*(*Θ_γ_*) is the adjustment coefficient. Considering that the high-frequency subbands in the three directions have the same importance, the same punishment coefficient is used.

According to discrete wavelet inverse transform, the image reconstructed by the scale coefficients is defined as *s*_1_ (*x*, *y*), the image reconstructed by the wavelet coefficients is denoted by *s*_2_(*x*, *y*), and the final reconstructed image ς(x,y) is defined as:

(33) $$\varsigma (x,y)=s_{1}(x,y)+s_{2}(x,y)$$

(34) $$s_{1}(x,y)=\frac{1}{\sqrt{MN}}\sum_{M}\sum_{N}W_{\phi }\left(j_{0},m,n\right)\varphi_{j_{0},m,n}(x,y)$$

(35) $$s_{2}(x,y)=\frac{1}{\sqrt{MN}}\sum_{i=H,V,D}\sum_{j=j_{0}}\sum_{M}\sum_{N}W_{\psi }^{i}(j,m,n)\psi_{j,m,n}^{i}(x,y)$$

**Figure 9:**
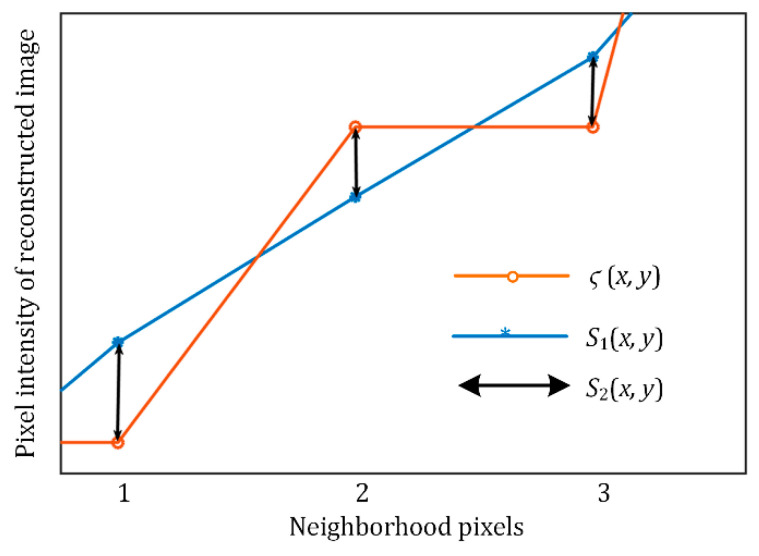
Relationship between adjacent pixels of ς(x,y), *s*_1_(*x*, *y*), s_2_(*x*, *y*).

Figure 9 shows the relationship between the images reconstructed by the scale coefficients and the wavelet coefficients. According to the correlation between adjacent pixels in the image, when the 3 neighboring pixels are on a straight line, the edge of the object can be considered smooth and not jagged; we define it as the edge smoothness preservation constraint, namely:

(36) 2×ς(x+1,y)=ς(x,y)+ς(x+2,y)

Substituting (33) into (36) yields:

(37) $$2\times [s_{1}(x+1,y)+s_{2}(x+1,y)]=[s_{1}(x,y)+s_{2}(x,y)]+[s_{1}(x+2,y)+s_{2}(x+2,y)]$$

The low-frequency coefficient after adaptive gamma correction is defined as *W_ϕ_*′(*j*_0_, *m*, *n*); the corresponding reconstructed image of *s*_1_′(*x*, *y*) is defined according to (34):

(38) $$\begin{array}{l} s_{1}{}^{\prime }(x,y)=\frac{1}{\sqrt{MN}}\sum_{M}\sum_{N}W_{\phi }{}^{\prime }(j_{0},m,n)\varphi_{j_{0},m,n}(x,y)=\frac{1}{\sqrt{MN}}\sum_{M}\sum_{N}I_{MAX}{\left(\frac{W_{\phi }(j_{0},m,n)}{I_{MAX}}\right)}^{\gamma }\varphi_{j_{0},m,n}(x,y)\\ =\frac{I_{MAX}{}^{1-\gamma }}{\sqrt{MN}}\sum_{M}\sum_{N}{\left(W_{\phi }(j_{0},m,n)\right)}^{\gamma }\varphi_{j_{0},m,n}(x,y) \end{array}$$

The gradient comparison of any pixel (*x_i_*, *y_i_*) between *s*_1_ (*x*, *y*) and *s*_1_′(*x*, *y*) is:

(39) $$\frac{\Delta s_{1}{}^{\prime }(x_{i},y_{i})}{\Delta s_{1}(x_{i},y_{i})}=I_{MAX}^{1-\gamma }\times {\frac{{\left(W_{\phi }(j_{0},m_{i},n_{i})+\Delta \right)}^{\gamma }-W_{\phi }(j_{0},m_{i},n_{i})}{\left(W_{\phi }(j_{0},m_{i},n_{i})+\Delta )-W_{\phi }(j_{0},m_{i},n_{i}\right)}}^{\gamma }\approx I_{MAX}^{1-\gamma }\times \gamma \times W_{\phi }{\left(j_{0},m_{i},n_{i}\right)}^{\gamma -1}$$

The high-frequency coefficients are adjusted by *L*(*Θ_γ_*) to obtain the reconstructed image *s*′_2_ (*x*, *y*):

(40) $$s_{2}{}^{\prime }(x,y)=\frac{L(\Theta_{\gamma })}{\sqrt{MN}}\sum_{i=H,V,D}\sum_{j=j_{0}}^{+\infty }\sum_{M}\sum_{N}W_{\psi }^{i}(j,m,n)\psi_{j,m,n}^{i}(x,y)=L(\Theta_{\gamma })\times s_{2}(x,y)$$

By substituting (39) and (40) into the edge smoothness preservation constraint (37), the punishment coefficient can be obtained:

(41) $$L(\Theta_{\gamma })=I_{MAX}^{1-\gamma }\times \gamma \times W_{\phi }{\left(j_{0},m_{0},n_{0}\right)}^{\gamma -1}$$

*L*(*Θ_γ_*) can adjust the high-frequency coefficient adaptively with *γ*(*Θ*_[*χ,σ*]_) to maintain the smoothness of the image edges.

### 3.6. Color Restoration

The HSV color model is used in our research because it is consistent with the human eye’s perception of color. It includes three characteristics: hue (H), saturation (S) and value (V). The V component represents the luminance intensity. GLAGC is performed on the V component. To restore the color information of the observed image, the output color image in RGB color space can be obtained by a linear transform [21], and the following improved operations are defined:

(42) $$\begin{array}{l} R{}^{\prime }(x,y)=\frac{V{}^{\prime }(x,y)}{V(x,y)+\zeta (x,y)}R(x,y)\\ G{}^{\prime }(x,y)=\frac{V{}^{\prime }(x,y)}{V(x,y)+\zeta (x,y)}G(x,y)\\ B{}^{\prime }(x,y)=\frac{V{}^{\prime }(x,y)}{V(x,y)+\zeta (x,y)}B(x,y) \end{array}$$

where *V*(*x*, *y*), *R*(*x*, *y*), *G*(*x*, *y*), and *B*(*x*, *y*) are the V, R, G, and B components before correction. *V*′(*x*, *y*), *R*′(*x*, *y*), *G*′(*x*, *y*), and *B*′(*x*, *y*) are the corresponding components after correction. *ζ*(*x*, *y*) is an adaptive stability factor that plays a role in low-illumination noise suppression, which is defined as:

(43) $$\zeta (x,y)=\beta \times \frac{V{}^{\prime }(x,y)}{V(x,y)}$$

where *β* is the adjustment coefficient. In general, *β* = 0.005.

To sum up, we describe the algorithm of the proposed GLAGC method in Algorithm 1.
**Algorithm 1** Algorithm for the adaptive dual-gamma function for image illumination perception and correction in the wavelet domain (GLAGC)**Algorithm’s inputs:** Original image *S*(*x*, *y*)**Algorithm’s output**: Enhanced image *O*(*x*, *y*)**Step (1)**:Convert to HSV space to obtain the **V** component**Step (2)**:Convert image to the logarithmic domain **v** = log(**V** + 1)**Step (3)**: Fast illuminance extraction in the *LL* subband by the wavelet transform**Step (4)**: Illuminance feature extraction:    Spatial luminance distribution feature (SLDF)    Global statistical luminance feature (*GSLF*)**Step (5)**: Adaptive dual-gamma correction *γ*(*Θ*_[*χ,σ*]_) for the *LL* subband    *γ*(*Θ_χ_*) (obtained by the *SLDF*)    *γ*(*Θ_σ_*) (obtained by the *GSLF* and Gamma training)**Step (6)**: Smoothness preservation *L*(*Θ_γ_*) for high-frequency coefficients**Step (7)**: Inverse wavelet and inverse logarithmic transform**Step (8)**: Color restoration

## 4. Experiments

During the experiments, first, the performances of LSAGC and GSAGC are verified. Next, image naturalness preservation through punishment adjustment and low-illumination noise suppression is illustrated. Then, the GLAGC method is qualitatively compared with several state-of-the-art methods. All the experiments are run in MATLAB R2017b for Windows 7 on a computer equipped with an Intel(R) Core (TM) i7-4790 CPU at 3.60 GHz and 8 GB memory. All the test images are sourced from related work [2,15,18,20,21,22,26] and benchmarks that have been commonly used for performance verification.

Four state-of-the-art algorithms were used for the comparison experiments, including the variational-based method SIRE [19], the AGCWD method combined with histograms [17], the 2-D adaptive gamma correction method (Sungmok Lee′s method) [18], and LIME based on Retinex theory [2]. All the parameters in the competing methods are chosen according to their original articles.

Four evaluation indicators were selected in the experiments:

(1) The computational cost of the algorithm;
(2) The information entropy, which is used to quantify and evaluate the information richness of the enhanced image;
(3) The absolute mean brightness error (AMBE) [27], which is used to evaluate illuminance retention, is defined as follows:
  (44) $$AMBE(x,y)=\left|x_{m}-y_{m}\right|$$
  where *x_m_* and *y_m_* represent the average value of the input image and output image, respectively.
(4) The lightness order error (LOE), which is used to evaluate the naturalness of image enhancement [26]:
  (45) $$\begin{array}{l} LOE=\frac{1}{mn}\sum_{i=1}^{m}\sum_{j=1}^{n}RD(i,j)\\ RD(x,y)=\sum_{i=1}^{m}\sum_{j=1}^{n}U(L(x,y),L(i,j))⊕U(L_{e}(x,y),L_{e}(i,j))\\ U(x,y)=\{\begin{array}{cc} 1 & x>y\\ 0 & else \end{array} \end{array}$$
  where *m*, *n* is the image size, *RD*(*i*, *j*) is the relative order of pixels (*i*, *j*), ⊕ is the exclusive or (XOR) operator, and *L*(*x*, *y*) and *L_e_*(*x*, *y*) are the original image and enhanced image, respectively. The smaller the LOE value is, the better the naturalness of the original image that can be maintained.

### 4.1. LSAGC Tests

This section will discuss the spatial distribution characteristics of different images and the influence of the proposed LSAGC function on the image spatial illumination distribution.

**Figure 10:**
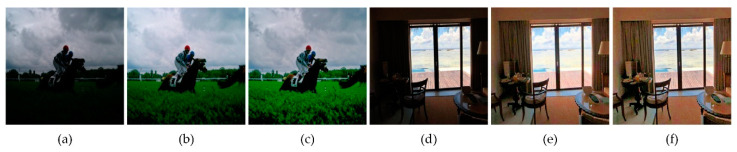
Effect of LSAGC. (**a**,**d**) Original images. (**b**,**e**) Enhanced images without LSAGC. (**c**,**f**) Enhanced images with LSAGC.

Figure 10 shows two images with uneven illumination distributions. The area where the lawn is located at the bottom of image (a) is in a weakly exposed state, and images (b) and (c) are the conditions without or with the LSAGC function, respectively. At the bottom of image (c), by LSAGC, the lawn becomes more obvious, and more detailed textures are also highlighted. Figure 10d shows the normal exposure of the sky in the middle of the image, and the indoor area next to it is weakly exposed. Without LSAGC, the sky area in the middle of the image becomes saturated after enhancement, resulting in the loss of texture and other information. When LSAGC is used, the texture of the sky is not overexposed, and information is not lost.

**Figure 11:**
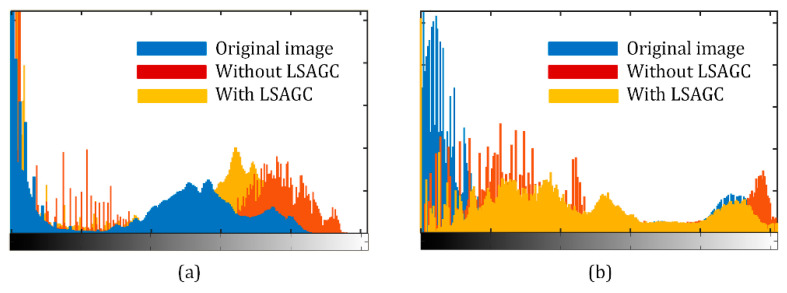
Histogram analysis: (**a**) images from Figure 10a–c; (**b**) images in Figure 10d–f.

It can be seen from the above two examples that LSAGC sufficiently considers the spatial characteristics of the illumination distribution and redistributes the uneven spatial illumination to make it more uniform. Histogram analysis is given in Figure 11. It can be seen that in the absence of LSAGC, more high pixel values will lead to overexposure; LSAGC can avoid this situation, and low-value pixels have also been better improved, improving the image illumination quality.

### 4.2. GSAGC Tests

**Figure 12:**
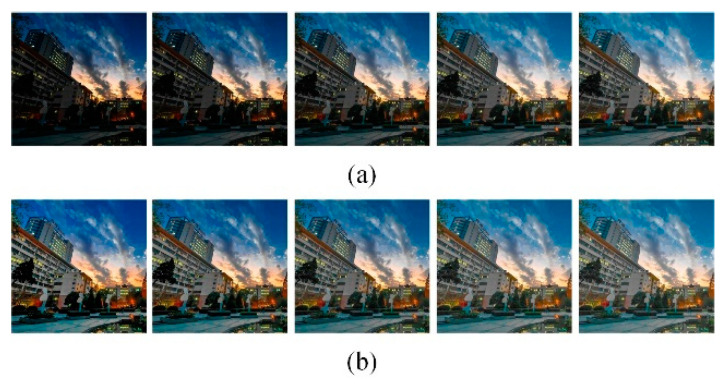
Park. (**a**) Input different global illumination image sequences. (**b**) Output almost the same global illumination image sequence.

**Figure 13:**
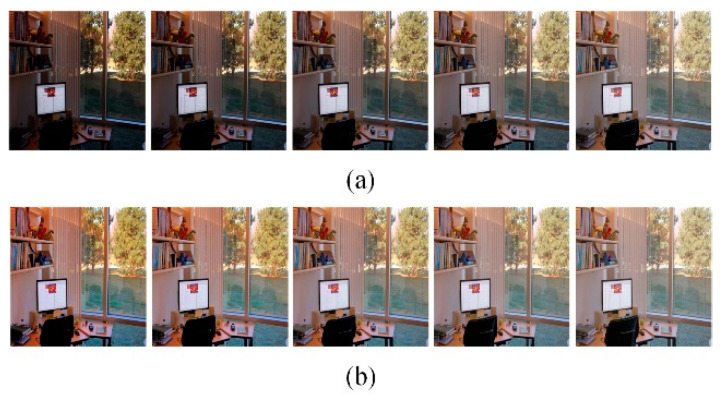
Office. (**a**) Input different global illumination image sequences. (**b**) Output almost the same global illumination image sequence.

**Figure 14:**
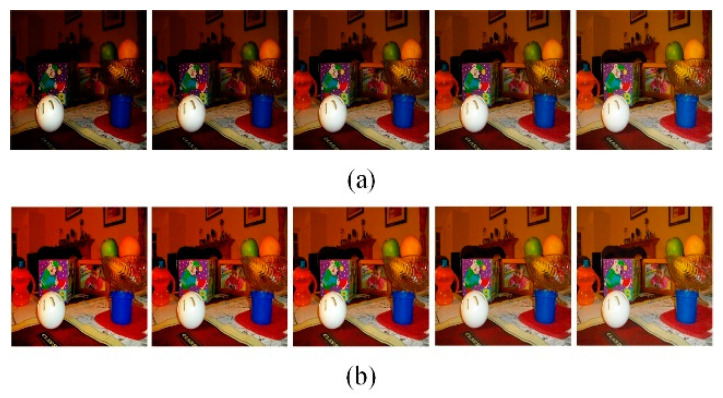
Flash toy. (**a**) Input different global illumination image sequences. (**b**) Output almost the same global illumination image sequence.

**Figure 15:**
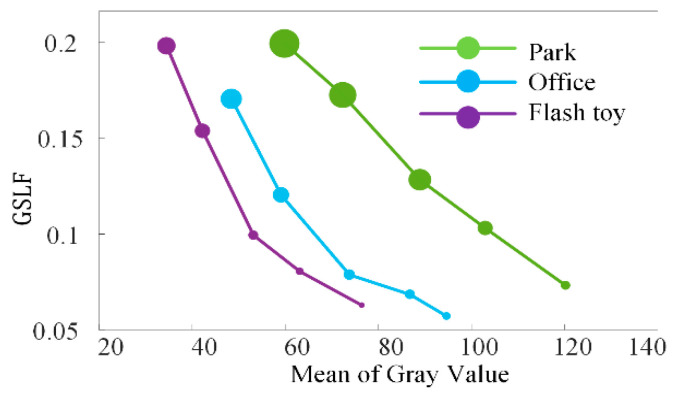
Relation of AMBE, *GSLF* and mean of gray value for the Park, the office, and the flash toy.

This experiment discusses the adaptive correction effect of the GSAGC method in the proposed algorithm on images with different global illumination values. Figure 12, Figure 13 and Figure 14 shows three sets of images, in which each experimental input sample is five images with different exposures from dark to bright, and we process them with the proposed algorithm, the parameters of which have been trained. The experimental results show that for images with different exposures, the proposed algorithm can automatically perceive the exposure level and generate high-quality images with almost the same exposure. Figure 15 uses the *GSLF* as the Y-axis of the above three sets of experimental input images and the mean of the image as the X-axis. The size of the circle indicates the AMBE value.

The *GSLF* defined in (26) is a measure of the global statistical illumination characteristics of an image. The larger the value is, the weaker the exposure. As shown in Figure 15, as the brightness of the input image gradually increases, the average value of the image gradually increases. When *GSLF* decreases, the exposure level is increasing, and the AMBE also subsequently decreases, indicating that the image brightness has been maintained and has not continued to increase when the image is properly exposed. This experiment shows that the proposed algorithm can enhance low-exposure images while maintaining normal-exposure images.

### 4.3. Naturalness Preservation

This section will explain the impact of adjustment on the high-frequency coefficients and the impact of adaptive stabilization factors on the image quality.

**Figure 16:**
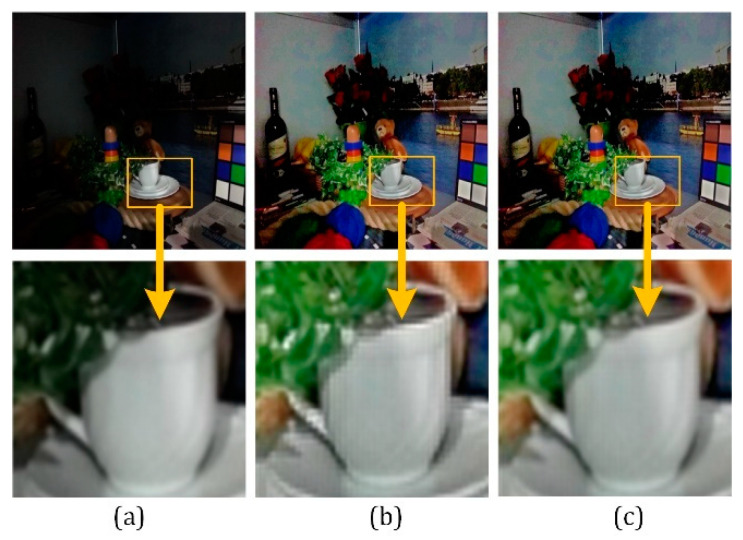
Effect of the smoothness preservation. (**a**) Original image. (**b**) Jagged image after enhancement. (**c**)Enhanced image with smoothness preservation.

Figure 16 shows the edge smoothness preservation test. When the high-frequency coefficients are not corrected, the edge smoothness of the image object will be destroyed. As shown in Figure 16b, when the adaptive adjustment is obtained when the edge smoothness preservation constraint is used, the image maintains the edge smoothness after being enhanced, improving the visibility of the images.

**Figure 17:**
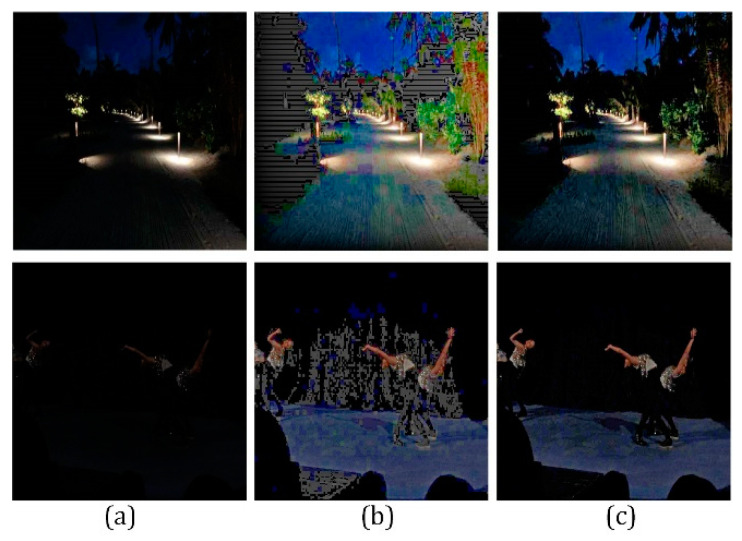
(**a**) Two original images; (**b**) Restored images without noise suppression; (**c**) Enhanced images with noise suppression.

Figure 17 illustrates the low-illumination noise suppression test. When the input image is extremely weakly exposed, as Figure 17b shows, considerable noise will appear at low-exposure areas after color restoration. By the adaptive stability factor defined in (43), the image quality is enhanced while noise is suppressed, as shown in Figure 17c.

### 4.4. Comparative Experiments

**Figure 18:**
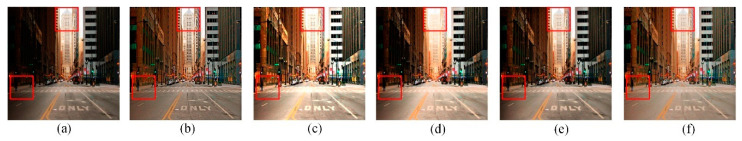
Urban image. (**a**) Original image; (**b**) result of Lee’s method; (**c**) result of LIME; (**d**) result of AGCWD; (**e**) result of SIRE; (f) result of the proposed method.

**Figure 19:**
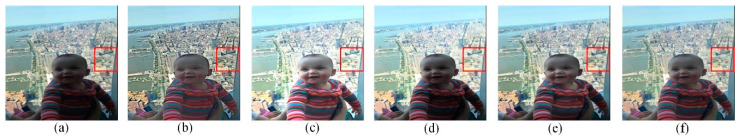
Baby image. (**a**) Original image; (**b**) result of Lee’s method; (**c**) result of LIME; (**d**) result of AGCWD; (**e**) result of SIRE; (**f**) result of the proposed method.

**Figure 20:**
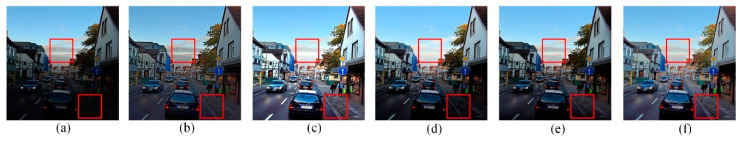
Street image. (**a**) Original image; (**b**) result of Lee’s method; (**c**) result of LIME; (**d**) result of AGCWD; (**e**) result of SIRE; (**f**) result of the proposed method.

We compare images under a series of illuminance scenarios through different algorithms, and the results obtained are shown below. As shown in Figure 18, Figure 19 and Figure 20, a group of images with uneven illumination distributions are called urban, baby, and street.

Figure 18 shows the experiments under the urban image. The AGCWD and SIRE methods cannot significantly enhance the dark areas surrounding the buildings. Moreover, the AGCWD method causes saturation on the upper part of the image; the LIME method can enhance the overall brightness of the image, but it causes overexposure; Lee’s method and the proposed method achieve good performances.

Figure 19 displays the results of the experiments on the baby image. The AGCWD and LIME methods both overenhance the background areas; moreover, the AGCWD method cannot increase the brightness in the baby’s clothes. The result from Lee’s method is overnormalized, and similar results are obtained by the LIME method and the proposed method.

Figure 20 presents the situation for the Street image, for which the best performance is achieved by our method. The AGCWD and SIRE methods cannot enhance the dark areas at the bottom of the image; the overall picture by Lee’s method is still dark; the LIME method seems to yield a bright image, but it causes oversaturation in the sky.

**Figure 21:**
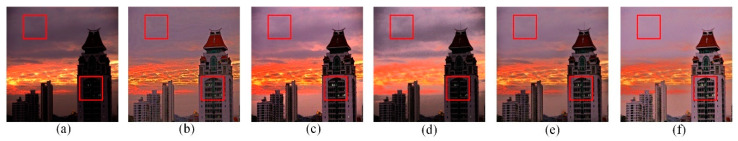
Buildings image. (**a**) Original image; (**b**) result of Lee’s method; (**c**) result of LIME; (**d**) result of AGCWD; (**e**) result of SIRE; (**f**) result of the proposed method.

**Figure 22:**
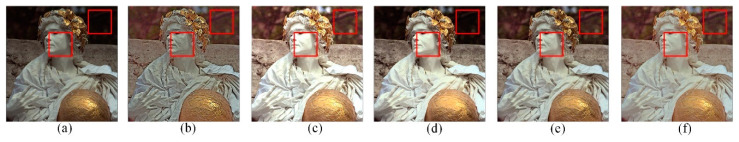
Goddess image. (**a**) Original image; (**b**) result of Lee’s method; (**c**) result of LIME; (**d**) result of AGCWD; (**e**) result of SIRE; (**f**) result of the proposed method.

**Figure 23:**
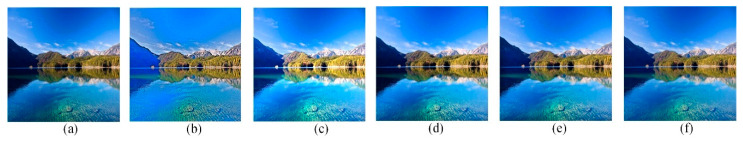
Landscape image. (**a**) Original image; (**b**) result of Lee’s method; (**c**) result of LIME; (**d**) result of AGCWD; (**e**) result of SIRE; (**f**) result of the proposed method.

The other image samples (composed of the building, goddess, and landscape images) have evenly distributed spatial illumination but with different global illumination, as shown in Figure 21, Figure 22 and Figure 23.

Figure 21 reveals the algorithms’ performance in complex lighting conditions. For shadow areas in the Buildings image in Figure 18. Our method outperforms all the other methods; the LIME and AGCWD methods cannot restore the colors of the dusk area; Lee’s method caused ripple distortions in the sky area.

Figure 22 is the comparison for the Goddess image. Lee’s method results in excessive contrast enhancement. Overenhancement is produced in the face region by the LIME and AGCWD methods; furthermore, the AGCWD method cannot remove the shadows in the background. The SIRE method achieves the best naturalness preservation, but the computational cost is much greater than that of our method, which will be discussed later.

Figure 23 shows the landscape image with pleasant visual effects, which are used to test the performance of avoiding overexposure. The mountain in the background becomes blue and loses its original color with Lee’s method; oversaturation occurs in the LIME method; the AGCWD and SIRE methods and our method all have good results.

**Figure 24:**
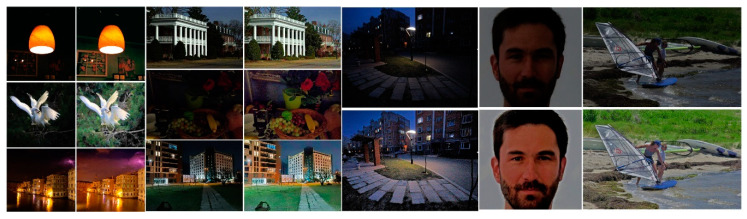
More results by our proposed GLAGC.

Figure 24 provides more experimental results by GLAGC. Table 1 shows the entropy, LOE and AMBE performance of the different algorithms. The proposed algorithm achieves the maximum value of the average information entropy of the enhanced image, which reveals that the proposed algorithm can obtain the most abundant image information. In terms of the preservation of naturalness, the proposed method has the lowest LOE after the AGCWD method, which is inferior to ours. Regarding the AMBE, the maximum is achieved by our method for dark illumination scenarios such as buildings, revealing its overall boosting on low illumination, while the minimum is obtained in normal-exposure scenes (Landscape), which presents brightness maintenance. Table 2 shows the average computational costs of the different algorithms under the same computational conditions, and the image resolution is 512 × 512. It can be seen that the proposed algorithm can achieve good results in a short amount of time.

In summary, by comparison experiments, the proposed method has good performance in low illumination enhancement, uneven illumination improvement and illumination maintenance. Lee’s method may cause ripple distortion and excessive contrast enhancement; the LIME method can handle the various illuminance conditions while the results will be oversaturated in some regions; the AGCWD method formulates the gamma mapping curve according to the histogram of the image without considering the spatial information, which results in degrading performance in uneven illumination images; and the SIRE method is relatively good. Nevertheless, its practicality is limited by time consumption.

## 5. Conclusions

In this article, we propose an adaptive image illumination perception and correction algorithm in the wavelet domain. We use the wavelet transform to obtain features of illuminance, and then the creative global statistical illuminance features and local spatial illuminance features are proposed as the foundation of perceived illuminance. An adaptive dual-gamma correction function is carried out accordingly; moreover, the edge smoothness is retained by adaptive adjustment. In addition, the proposed stabilization factor can suppress low-illumination noise. It is verified by comparative experiments that the adaptability, preservation of naturalness and efficiency of this algorithm on different images are improved compared with previous state-of-the-art methods. In addition to image enhancement, for a certain camera, our algorithm is promising for automatically providing an appropriate gamma factor through learning only several captured images.

## Figures and Tables

**Table 1:** Quantitative comparisons of different methods in terms of the entropy, LOE and AMBE.

Images	Index	LIME	Lee’s Method	AGCWD	SIRE	GLAGC
Urban	Entropy	7.59	7.62	7.67	7.72	7.78
	LOE	204.36	171.13	39.70	29.05	118.32
	AMBE	59.62	19.50	34.80	9.82	37.92
Baby	Entropy	7.09	7.77	7.67	7.83	7.76
	LOE	333.85	100.08	176.69	120.13	111.95
	AMBE	49.18	3.83	25.78	15.78	14.98
Street	Entropy	7.57	7.68	7.57	7.67	7.82
	LOE	282.43	93.54	89.56	141.58	173.6
	AMBE	56.03	15.48	24.46	18.29	39.15
Building	Entropy	7.54	7.11	7.50	7.42	7.35
	LOE	191.90	162.11	30.19	147.51	177.09
	AMBE	49.29	41.17	43.97	41.98	64.93
Goddess	Entropy	7.49	7.38	7.79	7.70	7.47
	LOE	199.21	283.96	43.77	192.93	105.01
	AMBE	72.12	19.35	44.42	34.17	43.87
Landscape	Entropy	7.83	7.64	7.78	7.46	7.82
	LOE	84.73	152.60	59.83	172.58	85.30
	AMBE	14.41	18.60	16.32	33.83	9.33
AVE.	Entropy	7.52	7.53	7.66	7.63	7.67
	LOE	204.36	138.24	66.04	127.58	115.88
	AMBE	50.111	19.66	31.62	27.31	35.03

**Table 2:** Average computational time (unit: seconds) of the different methods.

Lee’s Method	LIME	AGCWD	SIRE	Ours (GLAGC)
0.067	0.21	0.136	8.51	0.095

## Data Availability

Data sharing is not applicable. No new data were created or analyzed in this study.
